# Supplementary material for: Microsecond MD Simulations to Explore the Structural and Energetic Differences between the Human RXRα-PPARγ vs. RXRα-PPARγ-DNA
Source: Molecules. 2022 Sep 7;27(18):5778. doi: 10.3390/molecules27185778 (PMC9503000; doi:10.3390/molecules27185778)
Supplement: Supplementary file 1 [file molecules-27-05778-s001.zip › molecules-1882142-supplementary.pdf]

SUPPLEMENTARY MATERIAL

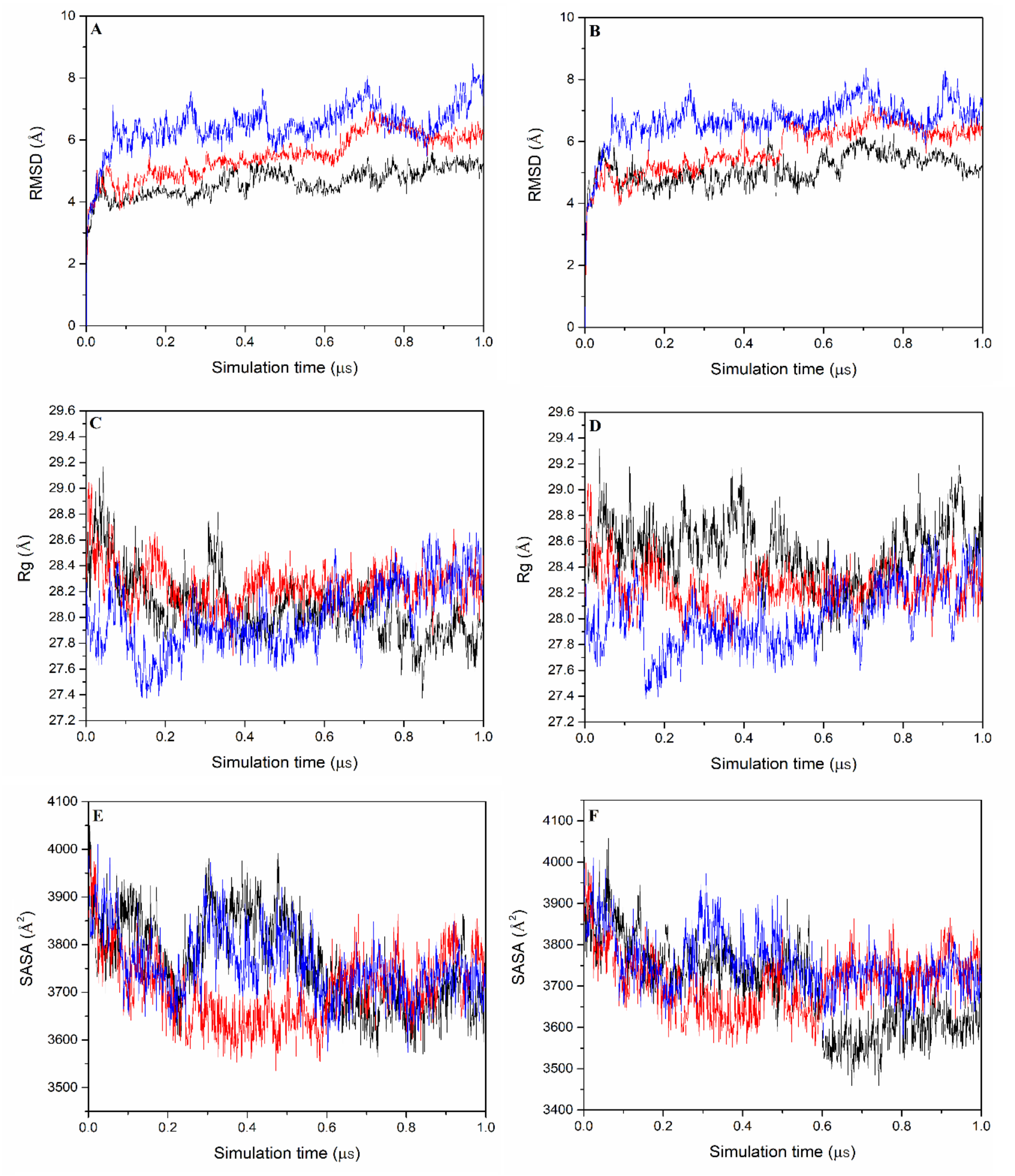

Figure S1. RMSD, RoG and SASA values of the PPARγ–RXRα–DNA and PPARγ–RXRα systems in complex with RZG and 9CR. RMSD values of the PPARγ–RXRα-DNA (A) and PPARγ–RXRα (B) systems. RoG values of the PPARγ–RXRα-DNA (C) and PPARγ–RXRα (D) systems. SASA values of the PPARγ–RXRα-DNA (E) and PPARγ–RXRα (F) systems. Black, red and blue lines represent the first, second and third simulation for each system.

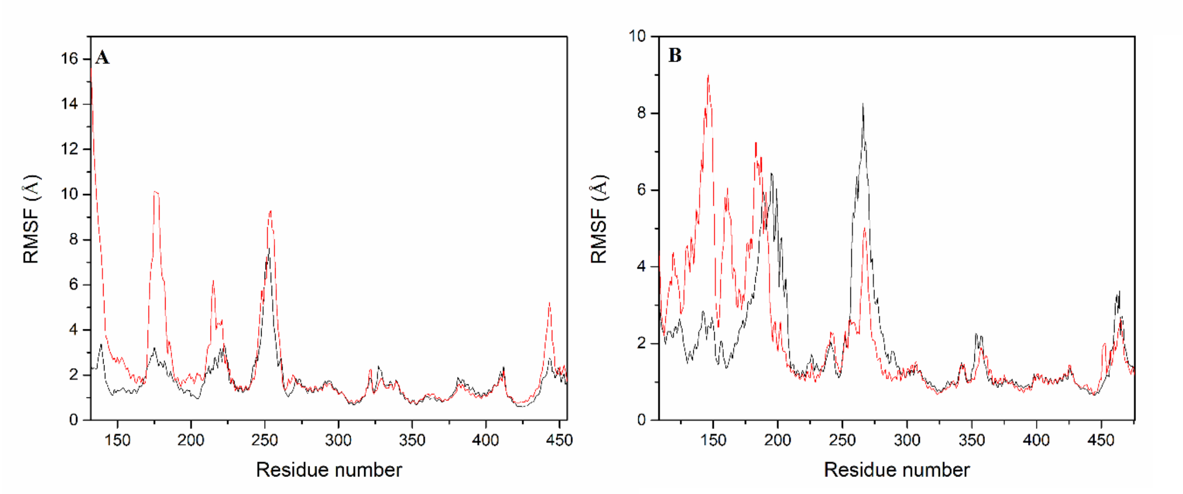

Figure S2. RMSF analysis of the PPARγ–RXRα–DNA and PPARγ–RXRα systems in complex with RZG and 9CR. A) RMSF values of the RXRα forming part of PPARγ–RXRα-DNA (black line) and PPARγ–RXRα (red line) systems. B) RMSF values of PPARγ forming part of PPARγ–RXRα-DNA (black line) and PPARγ–RXRα (red line) systems.

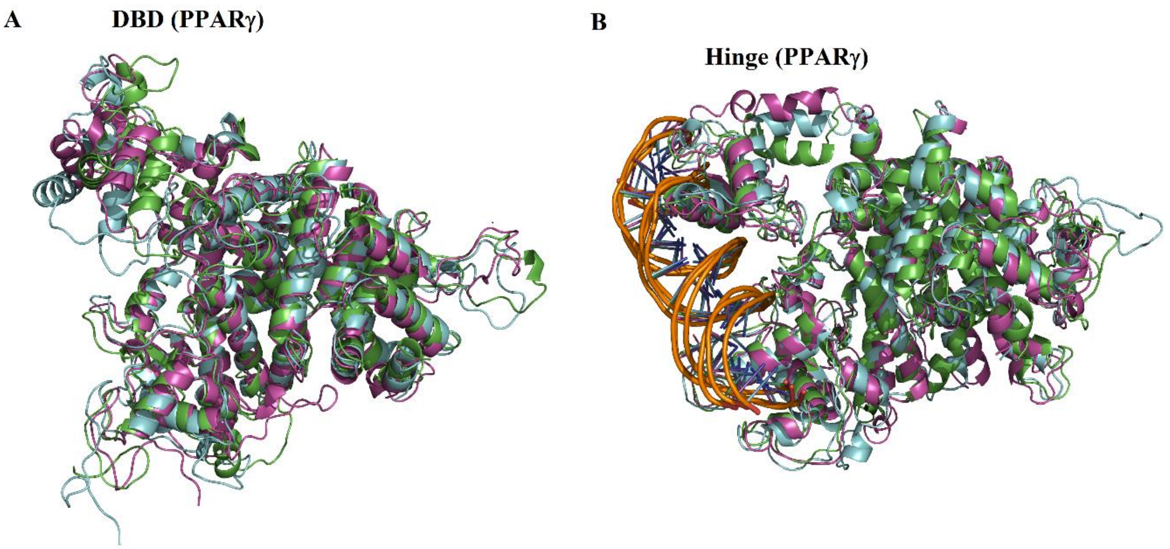

Figure S3. Overlapping of the three main conformations of PPARγ–RXRα and PPARγ–RXRα–DNA systems. Cartoon representation in green, cyan, and magenta colors correspond to the first, second, and third most populated conformation obtained through clustering analysis. The regions with significant structural differences are labeled in the figure.

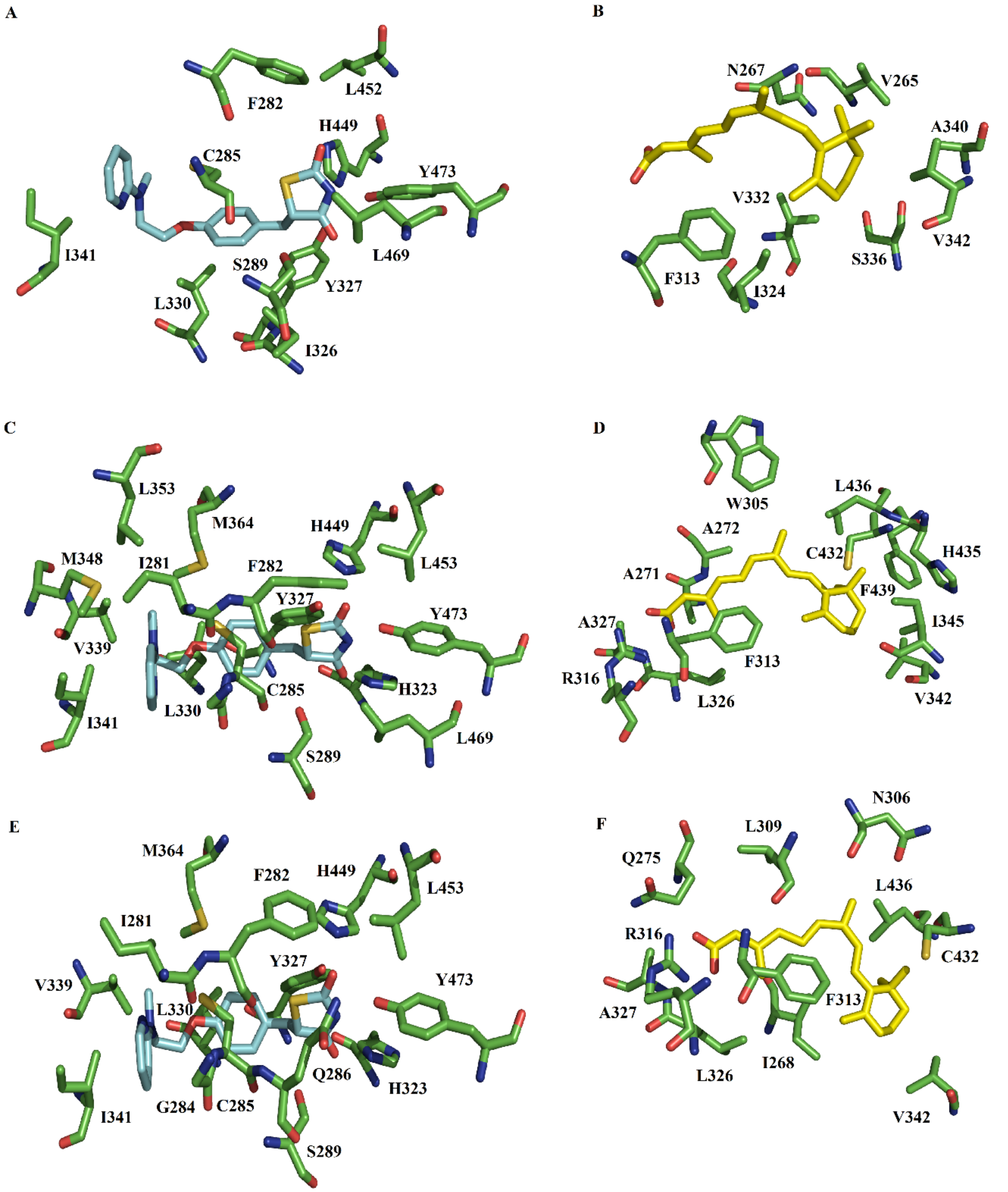

Figure S4. Binding mode of RGZ and 9CR in the crystallographic PPARγ–RXRα–DNA and PPARγ–RXRα complex. Interactions of RGZ (A) and 9CR (B) at the ligand-binding site of PPARγ–RXRα–DNA (PDB entry 3DZY). Contacts of RGZ (C) and 9CR (D) at the ligand-binding site of PPARγ–RXRα complex (PDB entry: 1FM6). Map of contacts of RGZ (E) and 9CR (F) at the ligand-binding site of PPARγ–RXRα complex (PDB entry: 5JI0)

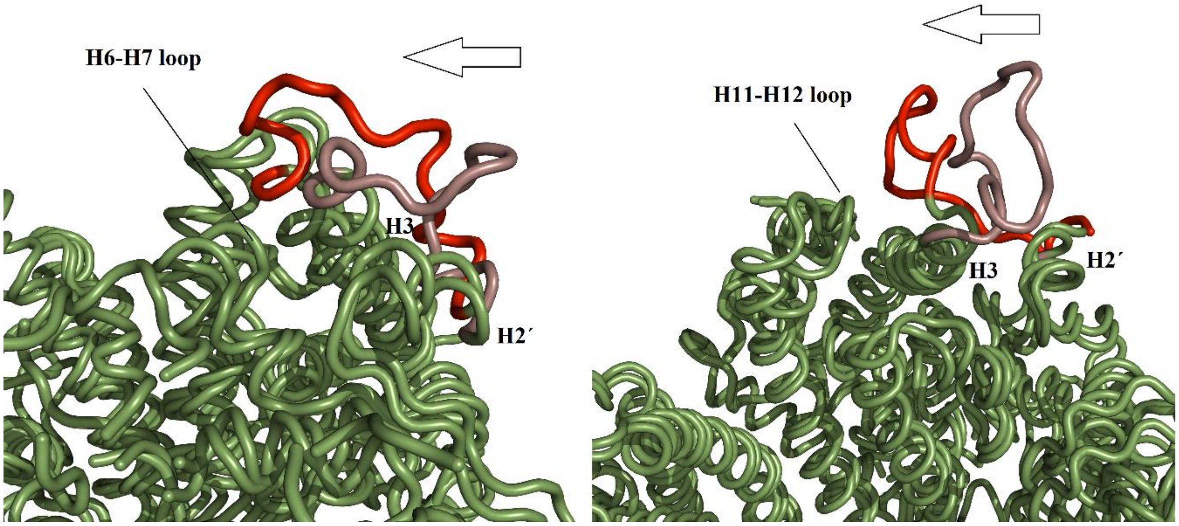

Figure S5. Movement of the H2'-H3 loop in PPARγ–RXRα (A) and PPARγ–RXRα–DNA (B) systems. The motion direction is by arrows. Dark salmon and red colors represent the initial and final motion.

Table S1. Percentage of conformations present in cluster analysis using RMSD cut-off of 3.5 Å.

**Cluster**	PPARγ–RXRα–DNA	PPARγ–RXRα
**1**	88.2	32.8
**2**	10.6	27.0
**3**	1.1	14.2
**4**	0.2	11.4
**5**		7.6
**6**		2.8
**7**		1.8
**8**		1.2
**9**		1.2

Table S2. Protein-protein interactions between RXRα and PPARγ for the RXRα–PPARγ, and RXRα–PPARγ–DNA systems.

RXRα-PPARγ-DNA (PDB entry 3DZY)	RXRα-PPARγ	RXRα-PPARγ-DNA
(DBD)ASP166.OD1-LYS336.NZ(β2-β4)		(DBD)ASP166.OD1-LYS336.NZ(β2-β4)
	(DBD)THR162.O-LYS336.NZ(β2-β4)	
	(DBD)TYR169.OH-GLU351.OE2(H6)	
	(DBD)ARG172.NH2-GLU369.OE2 (H7)	
	(DBD)LYS175.O-LYS358.NZ(H7)	
	(DBD)ASP176.O-LYS358.NZ(H7)	
	(DBD)ARG184.NH2-GLU351.OE2(H6)	
(DBD)TYR189.OH-GLU351.OE1(H6)	(DBD)TYR189.OH-ASP337.OD2(β2-β4)	(DBD)TYR189.OH-GLU351.OE1(H6)
(DBD)TYR192.OH-ASP337.OD2(β2-β4)	(DBD)TYR192.OH-ASP337.OD1(β2-β4)	(DBD)TYR192.OH-ASP337.OD2(β2-β4)
	(DBD)GLN193.NE2-ASP337.O(β2-β4)<br>(DBD)GLN193.OE1-ARG350.N(H6)<br>(DBD)GLN193.OE1-GLU351.N(H6)	
		(DBD)LEU196.CD1-PHE347.CE1(β2-β4)
(DBD)ARG202.NE-ASP337.OD2(β2-β4)	(DBD)ARG202.NH2-ASP337.OD1(β2-β4)	(DBD)ARG202.NE-ASP337.OD2(β2-β4)
(DBD)GLU203.OE1-ASN335.ND2(β2-β4)		(DBD)GLU203.OE2-ARG234.NE(H2)
(hinge)GLU207.OE2-LYS157.NZ(DBD)		(hinge)GLU207.OE2-LYS157.NZ(DBD)
	(hinge)ARG209.NE-GLU378.OE2(H7)	(hinge)ARG209.NH1-GLN163.OE1(DBD)
	(hinge)GLY212.O-LYS157.NZ(DBD)	
	(hinge)ASP214.OD1-LYS434.NZ(H10/11)	(hinge)ASP214.OD2-ARG153.NE(DBD)
	(hinge)ASN216.OD1-GLN430.NE2(H10/11)	
	(hinge)ASN218.O-ASN151.ND2(DBD)<br>(hinge)ASN218.ND2-SER428.O(H10/11)	
	(hinge)GLU219.OE1-ARG153.NH1(DBD)	
(H7)LYS356.NZ-GLU407.OE2(H9)	(H7)LYS356.NZ-GLU407.OE2(H9)	(H7)LYS356.NZ-GLY395.O(H8)<br>(H7)LYS356.NZ-GLU407.OE2(H9)
($Loop_{H8-H9}$)ASP379.OD2-LYS373.NZ(H7)	(L_{H8-H9})ASP379.OD1-LYS373.NZ(H7)	($Loop_{H8-H9}$)ASP379.OD2-LYS373.NZ(H7)
	(H9)GLU394.O-GLN430.NE2(H10/11)<br>(H9)GLU394.OE1-LYS434.NZ(H10/11)	(H9)GLU394.OE1-LYS434.NZ(H10/11)<br>(H9)GLU394.OE1-SER429.OG(H10/11)
	(H9)TYR397.OH-GLN437.OE1(H10/11)	
		(H9)GLU401.OE2-GLN430.NE2(H10/11)
		(H10/11)PHE415.CE2-PHE432.CD1(H10/11)

	(H10/11)LYS417.NZ-ASP411.OD1(H9)	(H10/11)LYS417.NZ-ASP411.OD1(H9)
(H10/11)LEU419.CD1-LEU436.CD1(H10/11)	(H10/11)LEU419.CD1-LEU436.CD1(H10/11)	(H10/11)LEU419.CB-LEU436.CD1(H10/11)
(H10/H11)LEU420.CD2-LEU436.CD2(H10/H11)<br><br>(H10/H11)LEU420.CD1-LEU414.CD1(H9)	(H10/11)LEU420.CD2-LEU414.CD1(H9)<br><br>(H10/11)LEU420.CD1-LEU436.CD2(H10/11)<br><br>(H10/11)LEU420.CD1-MET439.CE(H10/11)	(H10/11)LEU420.CD2-LEU414.CD1(H9)<br><br>(H10/11)LEU420.CD1-MET439.CE(H10/11)
	(H10/11)ARG421.NH1-ASP396.OD1(L_{H8-H9})	(H10/11)ARG421.NH1-ASP396.OD2($Loop_{H8-H9}$)
(H10/H11)ARG426.NE-ASP441.OD1(H10/H11)<br><br>(H10/H11)ARG426.NE-THR440.OG1(H10/H11)	(H10/11)ARG426.NH2-GLN444.OE1 (H10/11)	
	(H10/11) SER427.OG-THR447.OG1 (H10/11)	(H10/11)SER427.OG-THR447.OG1(H10/11)
		(H10/11)LYS431.NZ-GLN451.OE1(H10/11)<br><br>(H10/11)LYS431.NZ-TYR477.OXT(H12)
		(H10/11)GLU434.OE2-GLN451.NE2(H10/11)

Table S3. Per-residue free energy for the PPARγ–RXRα–DNA and PPARγ–RXRα systems coupled to RGZ and 9CR (values are presented in kcal/mol).

Residue	RXRα-PPARγ-DNA $PPARγ_{RSG}$	RXRα-PPARγ $PPARγ_{RSG}$	Residue	RXRα-PPARγ-DNA $RXRα_{9CR}$	RXRα-PPARγ $RXRα_{9CR}$
I262		-0.506	N262	-3.226	
I279	-0.412		D263	-0.282	
I281	-0.206	-0.492	P264	-1.621	-0.503
F282	-1.974	-0.607	V265	-0.258	-0.335
Q283	-0.643	-0.154	T266	-0.66	
G284		-0.842	N267		-1.006
C285	-1.181	-2.765	GLN270	-0.458	
			ALA272	-1.322	
Q286	-3.206	-1.313	LYS274	-0.108	
F287		-0.230	Q275	-2.501	
R288	-0.593	-1.726	L276	-1.779	
S289	-0.381	-0.807	T278	-0.317	
H323		-0.784	L279	-0.662	
I326	-1.096	-1.191	W305	-1.391	
Y327	-0.652	-1.316	N306	-0.153	
M329	-0.791	-0.140	L308	-0.161	
L330	-2.373	-1.623	L309	-1.242	
L333	-0.893	-0.419	I310		-0.449
V339	-0.297	-0.638	F313		-0.757
L340	-0.084		I324		-0.678
I341	-1.303	-2.386	L326		-1.503
S342	-0.7	-0.635	A327	-0.281	
E343	-1.035		L330		-0.318
M348		-0.477	V332		-1.061
L353		-0.166	S336		-0.138

L356	-0.152		A337		-0.671
F360	-0.643		A340		-0.653
F363	-0.819	-0.806	V342		-1.775
M364	-0.656	-0.610	I345		-1.238
K367	-0.369		F346		-0.666
H449	-1.179	-1.026	V349		-0.439
L452	-0.596		C432		-0.818
L453	-0.273	-0.197	H435		-0.433
I456	-0.451		L436	-0.603	-0.801
L465	-0.169		F439		-0.849
L469		-0.185	I447	-0.715	-0.850
Y473		-0.445	L451	-1.695	
			M454	-0.788	
			L455	-0.35	

Table S4. Per-residue free energy for protein-protein interactions of the RXRα–PPARγ–DNA, and RXRα–PPARγ systems coupled to RGZ and 9CR (values are presented in kcal/mol).

RXRα-PPARγ	Energy	RXRα-PPARγ-DNA	Energy
		(DBD) ASP166.OD1-LYS336.NZ (β2-β4)	-1.9
(DBD) THR162.O-LYS336.NZ (β2-β4)	-2.70		
(DBD) TYR169.OH-GLU351.OE2 (H6)	-6.30		
(DBD) ARG172.NH2-GLU369.OE2 (H7)	-0.90		
(DBD) ARG184.NH2-GLU351.OE2 (H6)	-5.90		
(DBD) TYR189.OH-ASP337.OD2 (β2-β4)	-2.60	(DBD) TYR189.OH-GLU351.OE1 (H6)	-0.3
(DBD) TYR192.OH-ASP337.OD1 (β2-β4)	-8.50	(DBD) TYR192.OH-ASP337.OD2 (β2-β4)	-1.0
(DBD) GLN193.NE2-ASP337.O (β2-β4)<br>(DBD) GLN193.OE1-ARG350.N (H6)<br>(DBD) GLN193.OE1-GLU351.N (H6)	-2.70		
		(DBD) LEU196.CD1-PHE347.CE1 (β2-β4)	-3.50
(DBD) ARG202.NH2-ASP337.OD1 (β2-β4)	-2.40	(DBD) ARG202.NE-ASP337.OD2 (β2-β4)	-5.80
		(DBD) GLU203.OE2-ARG234.NE (H2)	-2.00
		($L_{DBD-LBD}$) GLU207.OE2-LYS157.NZ (DBD)	-0.50
(DBD) ARG209.NE-GLU378.OE2 (H7)	-3.60	(DBD) ARG209.NH1-GLN163.OE1 (DBD)	-0.40
(DBD) GLY212.O-LYS157.NZ (DBD)	-0.60		
(DBD) ASP214.OD1-LYS434.NZ (H10/11)	-2.12	(DBD) ASP214.OD2-ARG153.NE (DBD)	-4.80
(DBD) ASN216.OD1-GLN430.NE2 (H10/11)	-4.40		
(DBD) ASN218.O-ASN151.ND2 (DBD)<br>(DBD) ASN218.ND2-SER428.O (H10/11)	-3.30		
(DBD) GLU219.OE1-ARG153.NH1 (DBD)	-3.90		
(H7) LYS356.NZ-GLU407.OE2 (H9)	-1.80	(H7) LYS356.NZ-GLY395.O (H8)<br>(H7) LYS356.NZ-GLU407.OE2 (H9)	-1.50
(H9) GLU394.O-GLN430.NE2 (H10/11)<br>(H9) GLU394.OE1-LYS434.NZ (H10/11)	-6.50	(H9) GLU394.OE1-LYS434.NZ (H10/11)<br>(H9) GLU394.OE1-SER429.OG (H10/11)	-3.90
(H9) TYR397.OH-GLN437.OE1 (H10/11)	-4.90		

		(H9) GLU401.OE2-GLN430.NE2 (H10/11)	-2.50
		(H10/11) PHE415.CE2-PHE432.CD1 (H10/11)	-1.30
(H10/11) LYS417.NZ-ASP411.OD1 (H9)	-1.95	(H10/11) LYS417.NZ-ASP411.OD1 (H9)	-1.90
(H10/11) LEU419.CD1-LEU436.CD1 (H10/11)	-2.00	(H10/11) LEU419.CB-LEU436.CD1 (H10/11)	-4.50
(H10/11) LEU420.CD2-LEU414.CD1 (H9)<br>(H10/11) LEU420.CD1-LEU436.CD2 (H10/11)<br>(H10/11) LEU420.CD1-MET439.CE (H10/11)	-2.70	(H10/11) LEU420.CD2-LEU414.CD1 (H9)<br>(H10/11) LEU420.CD1-MET439.CE (H10/11)	-4.80
(H10/11) ARG421.NH1-ASP396.OD1 (L_{H8-H9})	-5.50	(H10/11) ARG421.NH1-ASP396.OD2 (L_{H8-H9})	-7.30
(H10/11) ARG426.NH2-GLN444.OE1 (H10/11)	-2.00		
(H10/11) SER427.OG-THR447.OG1 (H10/11)	-1.20	(H10/11) SER427.OG-THR447.OG1 (H10/11)	-5.70
		(H10/11) LYS431.NZ-GLN451.OE1 (H10/11)<br>(H10/11) LYS431.NZ-TYR477.OXT (AF-2)	-6.00
		(H10/11) GLU434.OE2-GLN451.NE2 (H10/11)	-1.60
